# A deep auto-encoder model for gene expression prediction

**DOI:** 10.1186/s12864-017-4226-0

**Published:** 2017-11-17

**Authors:** Rui Xie, Jia Wen, Andrew Quitadamo, Jianlin Cheng, Xinghua Shi

**Affiliations:** 1 Department of Computer Science, University of Missouri at Columbia, Columbia, MO USA; 2 Department of Bioinformatics and Genomics, College of Computing and Informatics, University of North Carolina at Charlotte, University City Blvd, Charlotte, NC USA

**Keywords:** Predictive model, Stacked denoising auto-encoder, Multilayer perceptron, Deep learning, Gene expression

## Abstract

**Background:**

Gene expression is a key intermediate level that genotypes lead to a particular trait. Gene expression is affected by various factors including genotypes of genetic variants. With an aim of delineating the genetic impact on gene expression, we build a deep auto-encoder model to assess how good genetic variants will contribute to gene expression changes. This new deep learning model is a regression-based predictive model based on the MultiLayer Perceptron and Stacked Denoising Auto-encoder (MLP-SAE). The model is trained using a stacked denoising auto-encoder for feature selection and a multilayer perceptron framework for backpropagation. We further improve the model by introducing dropout to prevent overfitting and improve performance.

**Results:**

To demonstrate the usage of this model, we apply MLP-SAE to a real genomic datasets with genotypes and gene expression profiles measured in yeast. Our results show that the MLP-SAE model with dropout outperforms other models including Lasso, Random Forests and the MLP-SAE model without dropout. Using the MLP-SAE model with dropout, we show that gene expression quantifications predicted by the model solely based on genotypes, align well with true gene expression patterns.

**Conclusion:**

We provide a deep auto-encoder model for predicting gene expression from SNP genotypes. This study demonstrates that deep learning is appropriate for tackling another genomic problem, i.e., building predictive models to understand genotypes’ contribution to gene expression. With the emerging availability of richer genomic data, we anticipate that deep learning models play a bigger role in modeling and interpreting genomics.

## Background

As a critical biological process, gene expression represents a key intermediate level that genotypes could bring about effect on a particular phenotype. Changes in gene expression can result in phenotypic variation, while gene expression is manifested by many factors at various levels including genetic variants at DNA level. Hence, these genetic variants may influence phenotypes by potentially perturbing gene expression, and the fluctuations of gene expression could then give rise to an organism’s phenotypic changes. Genetic variants reflect the genetic difference among individuals and contain many types, ranging from single nucleotide polymorphisms (SNPs) to large structural variants. Recent sequencing initiatives have started to generate sequences of tens of thousands of individuals across a wide variety of species. For example, the Genome 10K Project [1] intends to assemble a “Noah’s Ark” of genomic data to help understand how complex animal life evolves and use this knowledge to save dying species. Thanks to the availability of these DNA sequences, the biological community has consequently generated detailed catalogs and genotypes of genetic variants in various biological systems. Studies have shown that genetic variants are associated with not only phenotypic traits of many kinds, but also linked with molecular traits such as gene expression. Therefore, assessing the effect of genetic variation on gene expression will improve our knowledge in understanding how genetic variation leads to phenotypic variation with regard to an organism’s development, growth and survival.

Quantitative trait locus (QTL) mapping has been demonstrated to be a powerful tool to associate genetic variation with quantitative traits. Particularly, expression QTL (eQTL) mapping [2, 3] has been widely performed to study the influence of genetic variants on gene expression, where gene expression is considered as a quantitative trait. Various eQTL studies [2] have been performed in yeast [4–17], zebrafish [18], human [19–31], and many other organisms. These eQTL studies have accumulated a growing list of SNPs associated with gene expression changes. Interestingly, SNPs associated with diseases through genome-wide association studies (GWAS) studies, are enriched for eQTLs [32, 33]. This observation points to an important perspective of understanding the link between genetic variants and gene expression, where eQTL studies can be employed to interpret and pinpoint GWAS findings especially for GWAS signals in non-coding regions.

Taking genotypes and gene expression quantifications as input, traditional eQTL mapping performs a statistical test (often using a linear regression or correlation model) between the genotypes of a genetic variant and the expression profiles of each gene in a set of samples. The nominal p-values from these statistical tests on all variant-gene pairs, will be subject to multiple test correction. Those variant-gene pairs passing statistical threshold for multiple test correction will be reported as significant associations. The majority of eQTL mapping is focused on *cis* analysis, where only local genetic variants located within a window of certain distance from the gene (typically using the coordinate of the transcription start site of the gene). Distal *trans* analysis, can be conducted in a similar fashion, where genetic variants outside of the designated window on the same chromosome or even on a different chromosome from the gene will be tested for associations.

However, genomic data for eQTL mapping is usually high-dimensional, where the numbers of genetic variants and genes are typically large and the sample size is relatively much smaller. Another feature of genomic data is the low signal-to-noise ratio, where only a very small amount of signals is relevant and the rest could be just noises. Given the sparsity and low signal-to-noise ratio of genomic data, it is thus statistically and computationally challenging to identify eQTL associations particularly *trans* associations. With the sparsity in mind, classical sparse learning methods, like the Least Absolute Shrinkage and Selection Operator (Lasso) model [34], can be used to identify associations between genetic variants and gene expression in eQTL mapping. This is because Lasso typically prefers solutions with fewer parameter values, and thus results in a sparse model which in turn make it appropriate to handle high-dimensional data as in genomic data analysis. Particularly, the Lasso model minimizes the usual sum of squared errors, with a bound on the sum of the absolute values of the coefficients. Thus, the Lasso model effectively reduces the number of variables upon which the given solution is dependent. For this reason, Lasso and its variants are fundamental to the field of compressed sensing. Under certain conditions, it can recover the exact set of non-zero weights. Therefore Lasso simultaneously produces an accurate yet sparse model, which makes it a feasible variable selection method for eQTL mapping.

One common critique of traditional eQTL mapping is that genetic variants and genes are tested independently. In reality, multiple genetic variants can be located within a haplotype block or sit on a pathway and hence their genotypes can be correlated. In the meanwhile, multiple genes can be co-regulated or involved in the same pathway and thus their expressions can be correlated. New methods have been proposed to incorporate the biological prior knowledge into eQTL mapping, such that the relationships between genetic variants and/or genes are taken into account. For example, multi-task Lasso and its graph-guided variants [14, 35–37] have been proposed for eQTL mapping. These graph-guided Lasso models work by adding regularization terms to a multi-task Lasso model, so that two variants or genes highly correlated are more likely to be selected together as a group.

Another approach previously used for eQTL mapping is Random Forests [38–41], which is an ensemble learning method for classification, regression and other tasks. By constructing a multitude of decision trees at training time, Random Forests are capable of performing classification or mean prediction (regression) of the individual trees. Additionally, Random Forests correct for decision trees’ habit of overfitting to their training set [42]. Studies have shown that Random Forests outperform traditional eQTL methods [43].

In addition to providing improved eQTL mapping, these machine learning based models have potentials for building a predictive model of inferring gene expression from genotypes. Note that this goal of prediction differs from the aforementioned eQTL mapping since eQTL mapping only focuses on constructing a mapping between genetic variants and associated genes, rather than predicting gene expression levels [44, 45]. Recently, a K-Nearest-Neighbor (KNN) method and a regularization linear regression model (i.e. Elastic Net) have been showed to allow for prediction of gene expression from only SNP genotypes in human Lymphoblastoid cell lines [44, 45]. Therefore, it is desirable to investigate innovative machine learning models and assess their capabilities of predicting gene expression from genotypes.

In addition to identifying eQTL associations, these machine learning models are well posed to be used for constructing predictive models. In this study, we set out to explore emerging deep learning models to build such a predictive model. Deep learning [46] has been demonstrated as a powerful model that shows encouraging performance in many tasks including text mining, natural language processing, image and video analysis [47]. Deep learning differs from previous shallow models in that they include a hierarchy of hidden layers that captures unknown structure in data. These hierarchical hidden layers, where higher levels represent more abstract entities, map the lowest input layer to the uppermost output layer without using hand-crafted features or rules [48]. With the rapid growth of genomics data, we witness an increase of deep learning models that encode hierarchical representations of various biological mechanisms captured in genomics data. For example, a deep neural network was developed that uses RNASeq data to predict splicing patterns in different tissues in mouse and evaluate differences in splicing patterns across tissues [49]. Another work built a convolutional neural network model to investigate the activities of transcription factors and histone modifications during E2-induced G1e differentiation [50]. Other examples of deep learning models in genomics include models to predict protein contact map [51, 52], protein residue-residue contacts [53, 54], protein sequence labeling [55], protein disorderedness [56, 57], protein structures [58–61], protein properties [62], protein fold recognition [63], the functional effect of non-coding variants [64], the pathogenicity of variants [65], and the regulatory code of genomes [66, 67].

Nonetheless, there is limited research with regard to predicting a quantitative trait from genetic variation. To investigate the feasibility of doing so, we develop a deep learning model to predict gene expression, a quantitative molecular trait, from solely genotypes of genetic variants in the same samples. Specifically, we construct a deep learning model based on MultiLayer Perceptron and Stacked Denoising Auto-encoder (MLP-SAE) to accommodate the high-dimensional genomic data. As seen in Fig. 2, this MLP-SAE model includes four layers, namely one input layer, one output layer and two hidden layers using stacked denoising auto-encoders. Each layer is pre-trained using a local unsupervised criterion. The model is further improved to prevent overfitting by using a dropout technique.

To assess the performance of the proposed MLP-SAE model, we compare it with other commonly used methods (e.g. Lasso and Random Forest) on real genomic datasets on yeast. We observe that our MLP-SAE model with dropout outperforms other models to predict gene expression patterns from solely genotypes of genetic variants on yeast. In summary, this study applies a deep learning model to address yet another biological problem, that is, predicting quantitative traits from genotypes for genomic prediction. This model is demonstrated to work well in predicting gene expression quantifications in yeast but can be applied to many other organisms to predict various traits not limited to gene expression.

## Methods

### Data collection and pre-processing

We collect a widely-used yeast data set, with 2 956 SNPs genotyped and the expression of 7 085 genes measured in 112 samples which are crosses of the BY4716 and RM11-1a strains [68]. We then remove missing values (denoted as ‘NA’) in the gene expression quantifications, resulted in the expression profiles of 6 611 genes. We pre-process the SNP genotype file by conducting imputing and scaling using the Imputer and MinMaxScaler [69] tookits in the Scikit-Learn package [70].

### Deep learning regression model

Since the output for gene expression prediction is quantitative, we use a linear regression model as the final layer of our deep learning model to generate the output. A linear regression model can be formalized as in Eq. 1.

1 $$ f(x) = \omega^{T} x + b.  $$

Here, *x* stands for the input variables or features (in this case, the genotypes of genetic variants), *y* represents the output or labels which are gene expression quantifications in this study, *w* is the weight matrix and *b* is the bias. In such a linear regression model, both *w* and *b* can be trained to minimize the objective function.

### Multilayer perceptrons

A Multilayer Perceptron (MLP) is a feedforward neural network that maps the input to the output. A MLP is composed of nodes (i.e. neurons) at multiple layers, including the input, output, and one or more hidden layers. Each layer in a MLP is fully connected with the next layer. In the hidden layers, each node is operated with a nonlinear activation function. Typically, two types of activation functions are used dependent on the data values operated on each node. Let’s use *o*_*i*_ to represent the output of the *i*th node, and *v*_*i*_ to represent the weighted sum of the input synapses. For a value within a range from 0 to 1, a logistic function is used as described in Eq. 2.

2 $$ o \left(v_{i} \right) = (1+e^{- v_{i}})^{-1}.  $$

For a value ranging from −1 to 1, a hyperbolic tangent is used as in Eq. 3.

3 $$  o \left(v_{i} \right) = tanh \left(v_{i}\right).  $$

After the data of each neuron in a MLP is processed, the MLP network can be learned by adjusting connection weights between nodes, using a backpropagation algorithm [71]. By comparing the output predicted by a MLP and the expected output values, we calculate the errors of the MLP and use supervised learning to learn a MLP model by minimizing the calculated error.

### Denoising auto-encoders

An auto-encoder [72] is another type of neural networks that helps learning efficient codings of input data. With a primary goal of learning a compressed and distributed representation (i.e. encoding) of the input data, an auto-encoder can thus be used for dimensionality reduction. Similar to a MLP, a simple form of an auto-encoder is a feedforward and non-recurrent neural net [73], which consists an input layer, an output layer and one or multiple hidden layers in between. In an auto-encoder, the activation of the final hidden layer can be treated as a compressed representation of the input, if the hidden layers have fewer nodes than the input or output layers. The activation functions used in a MLP can be also applied to auto-encoders.

Despite their similarities, an auto-encoder differs from a MLP in many ways. For example, the output layer of an auto-encoder has the same number of nodes as in the input layer. While an MLP can be learned to predict some target value *y* given the input *x*, an auto-encoder is trained to reconstruct its original input *x* by generating a reconstructed input *x* ^′^ through optimizing its objective function. For an auto-encoder, the model tries to reproduce the provided input data *x* by using supervised learning, where the difference between the original input *x* and reconstructed input *x* ^′^ is minimized. Therefore, backpropagation is also appropriate for training an auto-encoder [74].

The training process of an auto-encoded is usually based on backpropagation with the following three iterative steps. First, we perform a feedforward pass to compute the data values of all nodes after activation in the hidden layers and generate an output $\widehat {x}$ at the output layer for each input *x*. Second, we calculate the deviation of the output $\widehat {x}$ from the input *x* using measurements like square errors. Last, we backpropagate the calculated error through the network and update weights on the links using strategies like stochastic gradient descent algorithms.

**Fig. 1:**
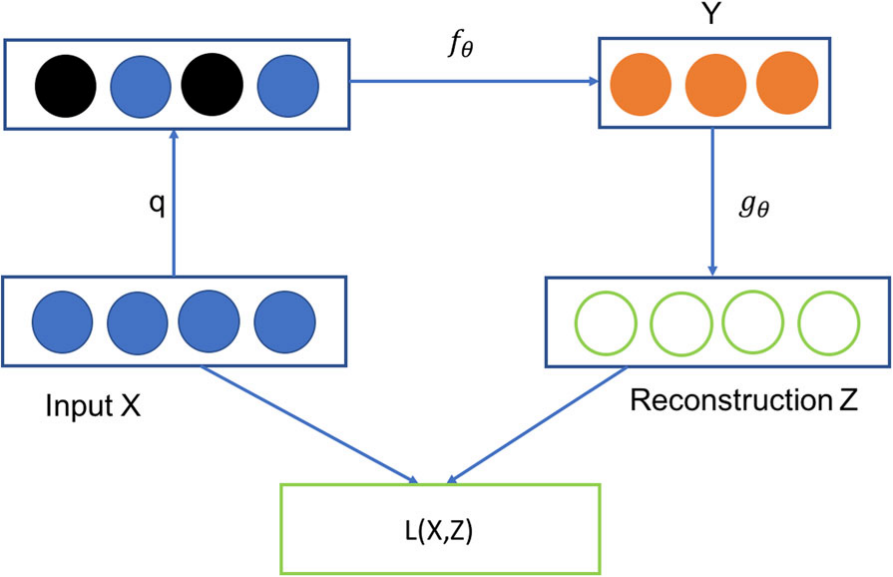
An Illustration of an Auto-encoder Corruption Model. The raw input *X* is corrupted via process *q*. The black nodes denote the corrupted input. The corrupted input is converted to *Y* via process *f*_*θ*_. Afterwards, *Y* attempts to reconstruct the raw input via process *g*_*θ*_, and generates the reconstruction *Z*. A loss function *L*(*X*,*Z*) is used to calculate the reconstruction error for backpropagation

To build robust models from high-dimensional data, a denoising auto-encoder has been developed as an extension of a classical auto-encoder [72]. The main goal of such a denoising auto-encoder is to separate signals from noises, which will allow the model to robustly reconstruct the output from partially destroyed input. Specifically, the corruption process of a denoising auto-encoder, as illustrated in Fig. 1, can be conducted in the following four steps. *Step 1:* A process *q* is performed to corrupt the input X is corrupted. *Step 2:* The corrupted input is mapped to *Y* via process *f*_*θ*_. *Step 3:* A process *g*_*θ*_ is conducted to reconstruct *Y* and generate the reconstruction of *Z*. *Step 4:* The reconstruction error is measured by a loss function *L*(*X*,*Z*), which will be used for backpropogation. The training process in a denoising auto-encoder is targeted for minimizing the loss function by resampling the shuffled inputs and re-reconstructing the data. The training of the model is completed when it finds the input that brings its model closest to the truth.

**Fig. 2:**
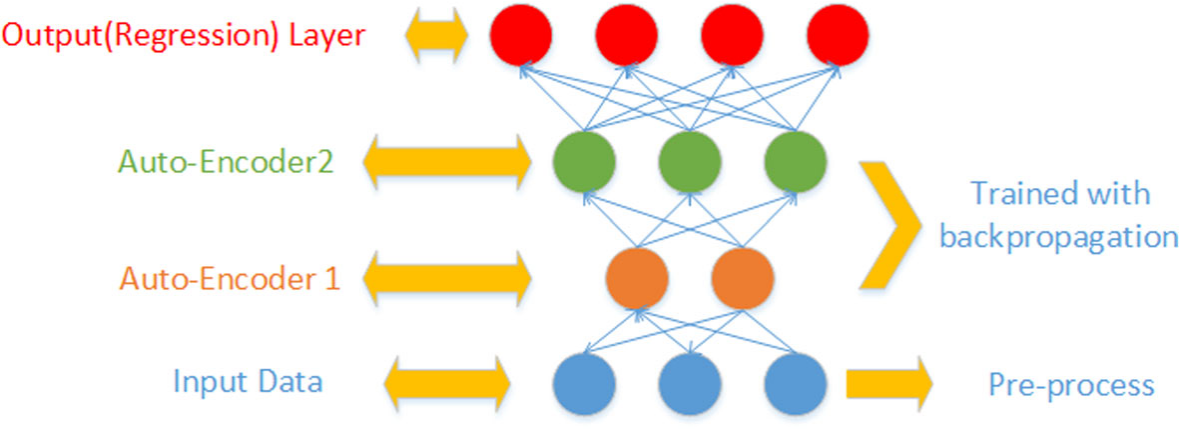
An Overview of Our MLP-DAE Model. The input layers takes the pre-processed data. The auto-encoder1 and auto-encoder2 serve as hidden layers for the prediction model and are trained using back propagation. The output layer is built on a regression model to make the final predictions

The loss function can be denoted as *L*(*X*,*Z*) between the original *X* and the reconstruction *Z* [75]. Different loss functions can be used. For example, a squared error objective can be used for a real value *X* as in Eq. 4) and a cross-entropy objective for a binary *X* as in Eq. 5)

4 $$  L \left(X,Z\right) = || X - Z ||^{2}  $$

5 $$  L \left(X, Z\right) = - \sum_{k}^{d} [x_{k} log z_{k} - \left(1-x_{k}\right) log (1-z_{k})]  $$

These denoising auto-encoders can be stacked as building blocks for constructing deep networks such as MLPs [72]. The performance of a traditional MLP is not good if we directly optimize a supervised objective function using algorithms like gradient descent with randomly initialized parameters. A better MLP can be constructed by applying a local unsupervised learning to pre-train each layer in turn, and produce a useful higher-level representation from the lower-level one using the output from the previous layer. By doing so, the gradient descent on the supervised objective leads to much better solutions in terms of generalization performance [75]. With this in mind, we use the MLP with stacked denoising auto-encoders and utilize pre-training and backpropogation in this study.

### The MLP-SAE model

To build a predictive model for estimating gene expression from genetic variation, we construct a deep denoising auto-encoder model utilizing the Multilayer Perceptron and Stacked Denoising Auto-Encoder (MLP-SAE). As shown in Fig. 2, our proposed MLP-SAE model is composed of four layers, one input, one output, and two hidden layers including two auto encoders. The input layer takes input as SNP genotypes from yeast, with pre-processing conducted before feeding into the model. The output layer of the model is a regression model which generates the output as the predicted gene expression values. Stacked denoising auto-encoders are used as the hidden layers of the model. The MLP-SAE model is trained and optimized by a backpropagation algorithm. The first training step is based on training the auto encoder with a stochastic gradient descent algorithm and the second training step utilizes the two auto-encoders as two hidden layers and training them with the multilayer perceptron. After training, we use cross validation to select the optimal model and evaluate the performance of the model on an independent data set.

**Fig. 3:**
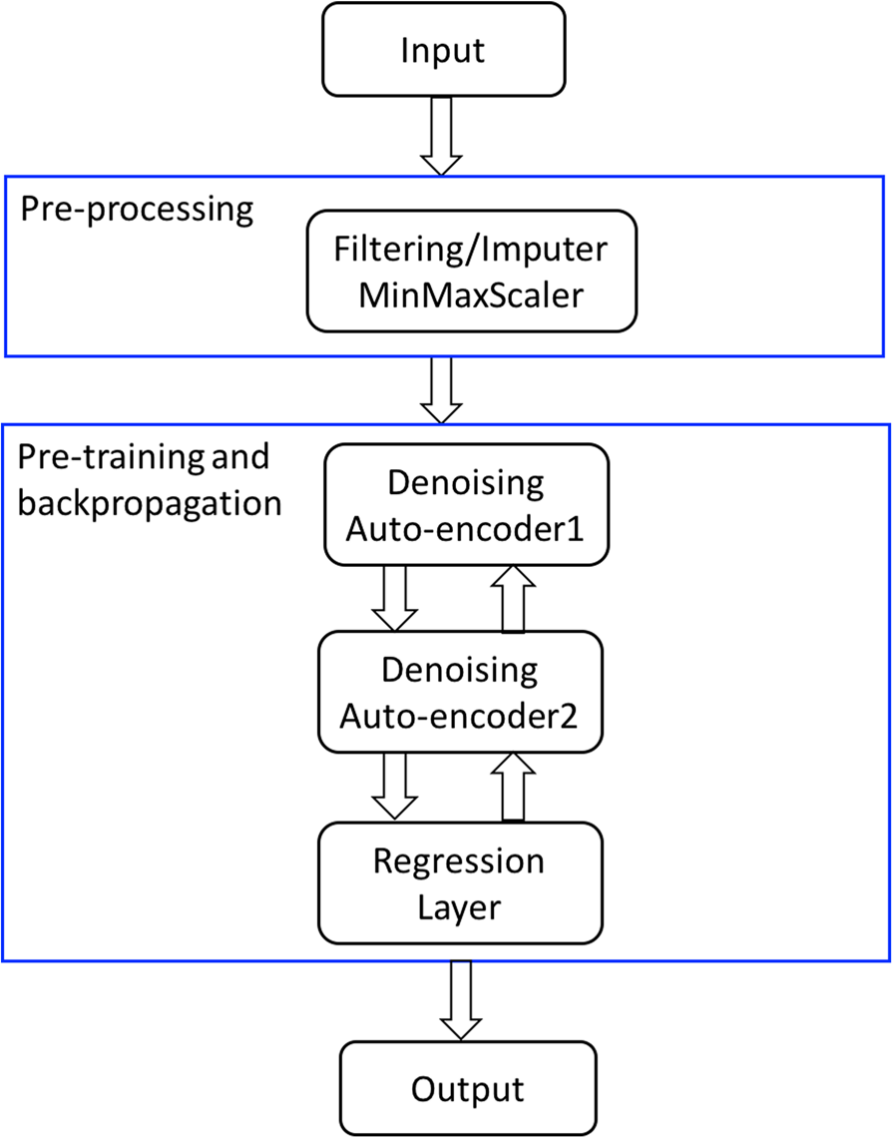
An Overall Workflow of the MLP-SAE Model. After pre-processing the input data, two layers of denoising autoencoders are used and the final regression layer produces the output of predicted gene expression quantification. The model is trained using pre-training and backpropagation for optimizing of the objective function

The detailed workflow of constructing this MLP-SAE regression model is illustrated in Fig. 3. The model first processes the raw input data and then performs pre-training as the first step of training. Next, the model is finetuned by backpropagation when it reaches the output layer. The algorithm stops when the model reaches convergence. The MLP-SAE model is implemented using the pylearn2 package [76].

### The MLP-SAE model with dropout

In modeling high-dimensional genomic model, overfitting is a challenging problem that needs to be carefully handled. One commonly-used strategy to prevent overfitting in a deep learning model is to apply dropout that has been shown to be able to efficiently combine many different neural network architectures [77]. In a neural network, the dropout strategy means that units are dropped out either at the hidden or visible layers to avoid overfitting and improve model performance. Specifically, to drop out a unit is to temporarily remove the unit from the network, along with all of its incoming and outgoing links in the network. A simple strategy for doing so is that each unit is kept in the network with a retention probability *p* independent of any other units. The probability *p* can chosen using a validation set, or naively set at 0.5. A pre-set of the probability *p* at 0.5, albeit simple, seems to be close to optimal for a variety of networks and tasks. One exception is that for the input units, the optimal probability of retention is usually close to 1 than 0.5.

With the above ideas in mind, a dropout strategy for a deep learning model, just like the application of regularization to mitigate overfitting works as follows. First, remove units and their associated weights in the network by a retention probability *p* for certain training samples and train the network with dropout using backpropagation. Second, repeat the dropout procedure (i.e., remove any other random set of units and their connections) and train the model on the training samples. Finally, take the mean of the weights across all of these modified network structures with dropouts when conducting predictions on new samples. In this study, we implement the MLP-SAE model on the yeast data with and without dropout respectively, and evaluate their performances.

### Other methods for comparison

We choose two methods to compare with our proposed MLP-SAE model, namely Lasso [34] and Random Forests. Previous study has shown that a regularization model using Elastic Net [44, 45] is capable of making predicted expressions that are highly correlated with observed expression values. We choose Lasso over Elastic Net because Lasso is sparse and fits well on high-dimensional genomic data. Lasso is a linear model with an *l*_1_ norm as regularizer, while a Elastic Net uses an *l*_2_ norm. As described in Eq. 6, the objective function of a Lasso model is to minimize the least-square penalty with an *l*_1_ norm.

6 $$  \min \frac{1} {2n} ||Xw - y||_{2}^{2} + \alpha ||w||_{1}.  $$

Here, *α* is a constant and ||*w*||_1_ is the *l*_1_-norm of the parameter vector. The hyperparameter *α* can be learned through training to control the sparsity of the model. When *α* is big, the model is sparser and more coefficients will be shrunk to zero with fewer features with non-zero coefficients being selected from the model.

In comparison, the Random Forests model is an ensemble method that has been shown to have nice prediction properties to solve a regression or classification problem [78]. Studies [79] have reported that Random Forests are related to KNN. KNN has been previously demonstrated as an efficient model to predict gene expressions from SNP genotypes [44, 45]. A Random Forests predictor has also been shown to outperform legacy eQTL methods in mapping genotypes to gene expression changes [43].

Therefore, in this study, we compare our newly developed MLP-SAE model with another two widely-used methods which have been shown to work well. Specifically, we evaluate Lasso, Random Forests, and MLP-SAE methods using the yeast dataset [68]. In our experimental setup, we split the dataset into three datasets, with a training dataset and validation dataset to be used in training phase, and an independent test dataset not part of any training to avoid overfitting. In addition, we extract part of the training dataset into a validation dataset, which does not participate in training, and then use five-fold cross validation on the training dataset to obtain the optimal model. Finally, we apply the trained model with learned parameters to an independent test dataset to obtain and compare the predictive results. To compare the performance of different models, we use mean square error (MSE, Eq. 7) for model evaluation.

7 $$  MSE = \frac{1} {n} \sum_{i}^{n} (z_{i} - y_{i})^{2}.  $$

Here, *n* is the number of samples, *y*_*i*_ is the original output, and *z*_*i*_ is the predicted output with *i* ∈ [1,*n*].

## Results

### MLP-SAE compared with Lasso and Random Forests

We first evaluate the performance of the three models respectively, namely Lasso, Random Forests and our newly developed MLP-SAE model. We conduct experiments on estimating the MSE values for each hyperparameter setting. Table 1 lists the hyperparameter learned for each model during training and resulted MSE values after cross validation. For Lasso, the hyperparameter learned via training is *α*, which controls the model sparsity. For Random Forests, the hyerparameter is the number of estimators or trees. For the MLP-SAE model, the hyperparameter determined in training is the learning rate. The row highlighted in bold in Table 1 shows the hyperparameter learned for the optimal model of Lasso (*α*=0.7, MSE=0.2912), Random Forests (the number of estimators=200, MSE=0.2967) and MLP-SAE (learning rate=0.1, MSE=0.2890) respectively using cross validation on the yeast data. With such optimal settings, we observe that the MLP-SAE model outperforms other classical methods like Lasso and Random Forests.

**Table 1:** Comparison of Lasso, Random Forests, and MLP-SAE model

Method	Hyperparameter	Hyperparameter value	MSE
Lasso	*α*	0.05	0.3516
		0.1	0.3182
		0.2	0.3002
		0.3	0.2951
		0.4	0.2930
		0.5	0.2918
		0.6	0.2914
		0.7	**0.2912**
		0.8	0.2912
Random forests	Number of estimators	10	0.3221
		20	0.3127
		30	0.3080
		40	0.3001
		50	0.2989
		60	0.3003
		70	0.2986
		100	0.3003
		150	0.2974
		**200**	**0.2967**
MLP-SAE model	Learning rate	**0.1**	**0.2890**
		0.01	0.2909
		0.001	0.2895
		0.0001	0.2908
		0.00001	0.2918

Each row represents the hyperparameter used and corresponding MSE for each hyperparameter setup of each model. Bold rows denote the hyperparameters and corresponding MSE for the optimal models of the three methods respectively

### MLP-SAE with and without dropout

We further improve the MLP-SAE model by incorporating dropout [77] to handle overfitting. Overfitting is a critical problem for high-dimensional data analysis, since there are typically more features/variables than samples in such data. We observe an improvement of performance of MLP-SAE with dropout, compared with MLP-SAE without dropout. The average MSE of MLP-SAE with dropout is 0.3082, while the average MSE of MLP-SAE without dropout is 0.3093.

**Fig. 4:**
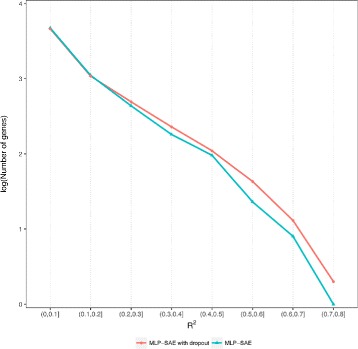
Predictions Using the MLP-SAE Model with Dropout are More Correlated with the True Gene Expressions than Predictions from the MLP-SAE Model Without Dropout. X axis denotes the correlation bins between true and predicted gene expression values, and Y axis represents the log of number of genes in each correlation bin

We then calculate the correlations (e.g. *R* ^2^ values) between the estimated expression and the true expression of each gene in the samples for both the MLP-SAE model and the MLP-SAE dropout model. Table 2 shows that there are more genes with higher correlations from MLP-SAE with dropout compared with the MLP-SAE model without dropout. As further illustrated in Fig. 4, there are more genes that are highly correlated between true expression values and estimated values using the MLP-SAE model with dropout than those predicted from the MLP-SAE model without dropout. In other words, the MLP-SAE model with dropout improves on the MLP-SAE model without dropout by making predictions that are more correlated with the true gene expression.

**Table 2:** Number of Genes Within *R* ^2^ Bins for MLP-SAE and MLP-SAE with Dropout Models

*R* ^2^	MLP-SAE	MLP-SAE with Dropout
(0,0.05]	3621	3507
(0.05,0.1]	1128	1121
(0.1,0.2]	1111	1086
(0.2,0.3]	436	493
(0.3,0.4]	181	229
(0.4,0.5]	96	110
(0.5,0.6]	23	43
(0.6,0.7]	8	13
(0.7,0.8]	0	2

For each gene, *R* ^2^ is calculated between the true and estimated expression values using the MLP-SAE model or the MLP-SAE model with dropout

Therefore, the MLP-SAE model with dropout has been demonstrated as the best model for predicting gene expression from SNP genotypes, based on our evaluations of three relevant models (e.g. Lasso, Random Forests, and MLP-SAE) on the yeast dataset. Earlier studies have shown that genomic features like functional annotations of the SNPs can be incorporated into a model to improve its predictive performance [44, 45]. Since our MLP-SAE model with dropout is based on deep learning, naturally it can be extended to incorporate evidence from other datasets or features including information on epigenetic markers and functional elements, and it should be scalable to larger datasets.

### Final results using MLP-SAE with dropout

**Fig. 5:**
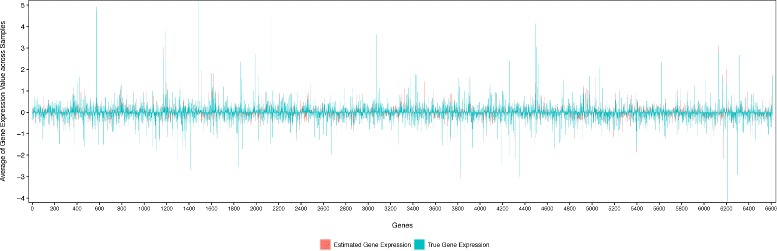
True Expression and Predicted Expression of All Genes Using MLP-SAE with Dropout

**Fig. 6:**
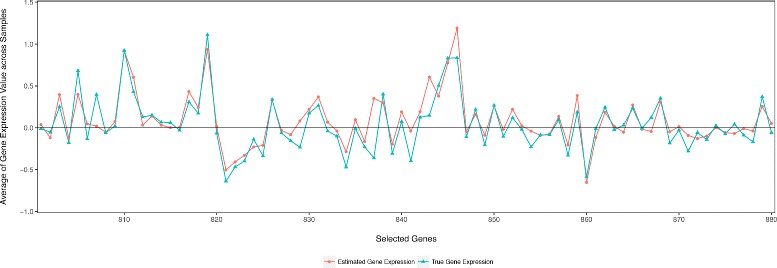
True Expression and Predicted Expression of Selected Genes Using MLP-SAE with Dropout

Using the best performance model in this study, i.e. the MLP-SAE model with dropout, we produce the final predictions of gene expressions solely from SNP genotypes on the yeast data. Figure 5 visualizes the true gene expression quantifications and the estimated values predicted from the model for all the 6 611 genes in the yeast data. Figure 6 zooms into a detailed view of the expression profiles of genes that are well predicted by our model. We observe that the estimated gene expression values predicted using our model align well with the true data. Although the true and estimated values are not always the same, our model recapitulates the changes in gene expression quantifications. In particular, the estimated gene expression values show similar peaks to the true values, while the absolute values might differ. This suggests that the gene expression estimations predicted using only SNP genotypes, encode similar up-regulated and down-regulated trend of gene expression compared to those expression profiles measured from gene expression microarrays as in the yeast data. Such observations are useful especially for those situations that gene expressions are not directly measurable. In those situations, we can use SNP genotypes to infer gene expression values to investigate the gene expression and regulation patterns, which are otherwise unavailable. Follow up analysis can then be conducted on the predicted gene expression quantifications to assess differential gene expression patterns under different conditions or across various tissues. Since genes are regulated at many different layers and controlled by multiple factors, we argue that our model can be further improved to recapitulate the true gene expression signals by including more features (e.g. annotation and sequences of regulatory elements, biological pathways and networks) and environmental conditions (e.g. diet, biomass production, growth/survival and temperature).

## Discussion

In this study, we demonstrate a case of using genotypes of SNPs to predict gene expression in yeast. Since genes are regulated at many different layers and controlled by multiple factors, we argue that our model can be further improved to recapitulate the true gene expression signals by including more types of features at different levels of gene transcription and regulation. For example, the annotations and sequences of regulatory elements can be included to leverage the contribution of variants in these regions for better gene expression prediction. Moreover, biological pathways networks can be incorporated in various forms such as regulatory networks, co-expression networks, signaling networks, and protein protein interaction networks. Since gene expression is dependent on environmental conditions (e.g. diet, biomass production, growth/survival and temperature) and tissue/cell types, such information should be also included in modeling gene expression. Additionally, gene expression can be affected by epigenetic markers including non-coding RNAs (e.g. microRNAs, long non-coding RNAs), DNA methylation, and histone modifications in many organisms. Such comprehensive epigenetic features can also be incorporated to help gene expression prediction.

This study uses a dataset with 2956 SNPs and 6611 genes assessed in 112 yeast samples. We anticipate that a larger dataset has potentials to capitalize the power of deep learning models to improve the modeling of gene expression. For instance, with the recent advance of single cell sequencing at DNA and RNA levels, we expect to gain an unprecedented amount of data for predicting gene expression (and other outcomes) from genomic sequences and genotypes.

## Conclusion

In this study, we provide a new deep learning model based on a deep denoising auto-encoder, namely the Multilayer Perceptron with Stacked Denoising Auto-encoder (MLP-SAE), for predicting gene expression profiles from genotypes. Applying the MLP-SAE model with dropout to a well-established yeast dataset [68], we show that this model outperforms other models including MLP-SAE without dropout, Lasso and Random Forests. In addition to its nice properties, the MLP-SAE model with dropout can be extended to include many other data types (e.g. epigenetic, metabolic, and environmental factors) to further improve the model performance. For example, protein quantifications [80, 81], metabolite screening [82, 83] and chromatin accessibility data [84, 85] are available for yeast. More comprehensive assessment of SNP genotypes and gene expressions of a larger cohort of yeast is also available [86, 87]. Such data can be incorporated into our model to predict any trait of interest, not limited to gene expression. Additionally, since the hierarchical layers of the MLP-SAE model with dropout can accommodate non-linear relationships in the input data, our model will naturally encapsulate complex interactions and structures encoded in the data. Therefore, our model can potentially capture epistasis and interactions, which have been shown to improve the modeling of quantitative traits of yeast [86, 87].

Although we focus on the yeast data set, our model is applicable for many other organisms. For example, a MLP-SAE with dropout model can be constructed to predict gene expressions in each tissue using the genotypes in the corresponding tissue, and then compare with true gene expression measurements to assess the model’s performance in recapitulating tissue-general and tissue-specific gene expression patterns in The Genotype-Tissue Expression (GTEx) project [88]. Additionally, there are many deep learning architectures such as the Restricted Boltzmann Machine [89] and Recurrent Neural Network [90], that can be applied to solve the quantitative trait prediction problem in this study. We anticipate that with the availability of richer data of more types, deep learning models have potentials to revolutionize genomic studies as in other fields.

## Abbreviations

eQTL: Expression quantitative trait locus
KNN: K-nearest-neighbor
Lasso: Least absolute shrinkage and selection operator
MLP: Multilayer perceptron
SAE: Stacked denoising auto-encoder
SNP: Single nucleotide polymorphisms
